# Supplementary material for: Targeting SUMO2 reverses aberrant epigenetic rewiring driven by SS18::SSX fusion oncoproteins and impairs sarcomagenesis
Source: EMBO J. 2025 Aug 13;44(18):4984–5004. doi: 10.1038/s44318-025-00526-w (PMC12436642; doi:10.1038/s44318-025-00526-w)
Supplement: Supplementary file 3 — Appendix [file 44318_2025_526_MOESM3_ESM.pdf]

Appendix for SUMO2 Inhibition Reverses Aberrant Epigenetic Rewiring Driven by Synovial Sarcoma Fusion Oncoproteins and Impairs Sarcomagenesis

Appendix Figure S1: 2

Appendix Figure S2: 3

Appendix Figure S3: 4

Appendix Figure S4: 5

Appendix Figure S5: 6

Appendix Figure S6: 8

Appendix Figure S7: 8

Appendix Figure S8: 9

Appendix Figure S9: 10

Appendix Figure S10: 11

Appendix Figure S11: 12

Appendix Figure S12: 13

Appendix Figure S13: 13

Appendix Figures:

S1

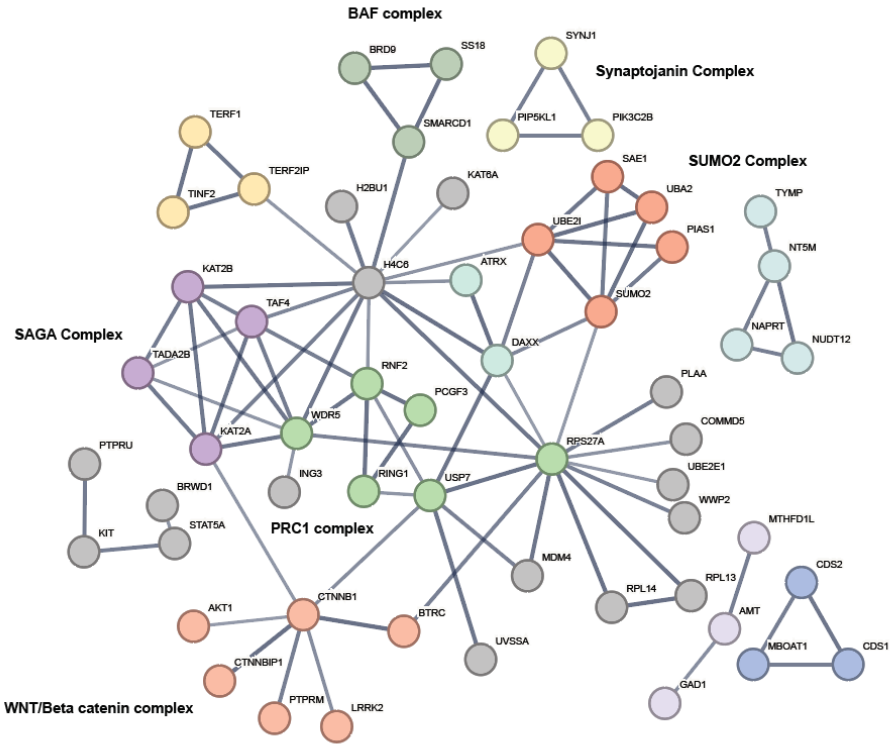

Appendix Figure S1:

String analysis of synovial sarcoma selective genes identified in our study is shown. Nodes indicate genes, edges indicate connections between the genes as represented by participation in common protein complexes identified in experimental data deposited from other groups.

S2

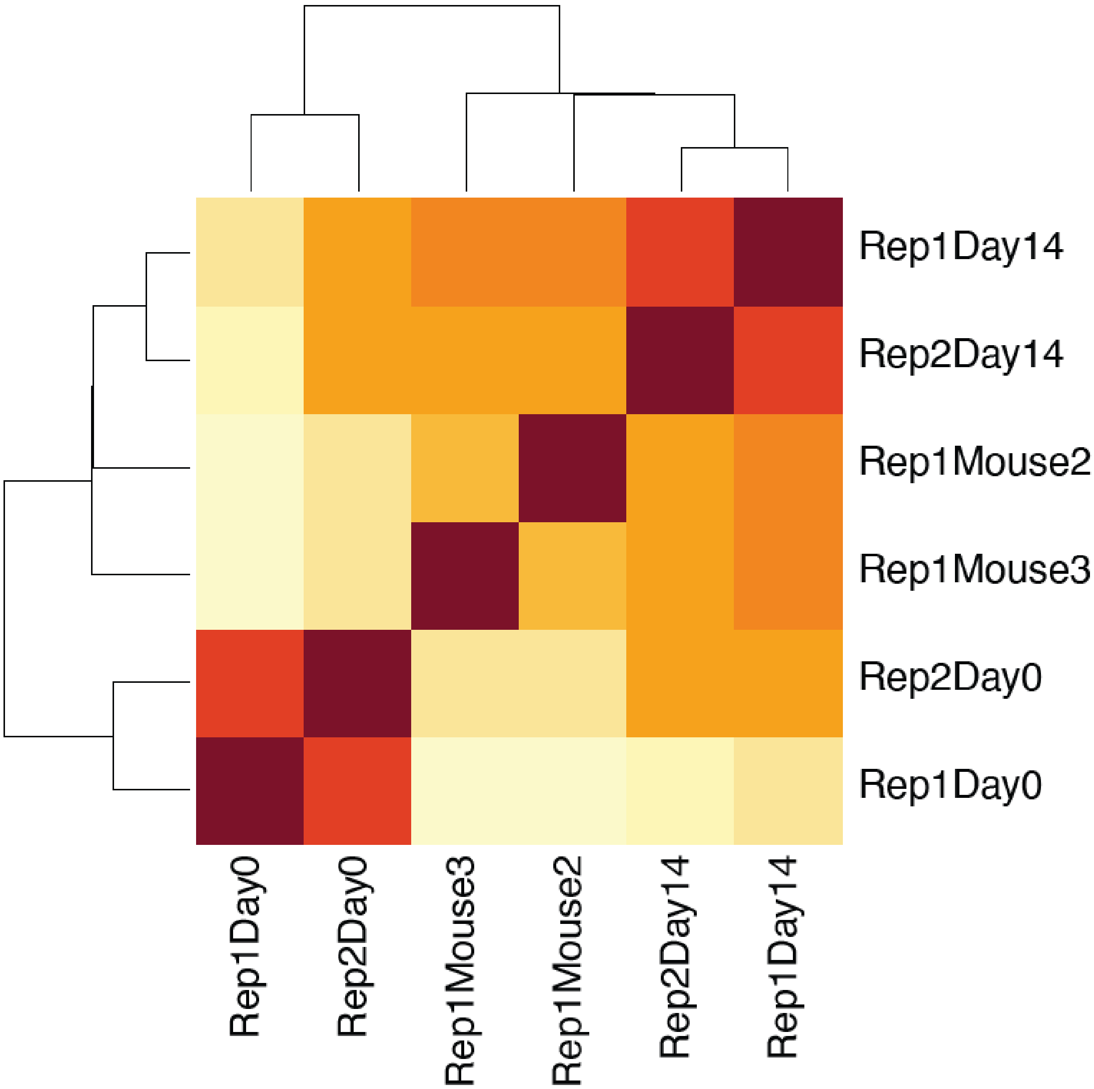

Appendix Figure S2:

Correlation matrix of replicates from the *in vitro* and *in vivo* screen is shown. Rep = replicate. Day 0 = start of the assay and D14 is the end of the *in vitro* assay.

S3

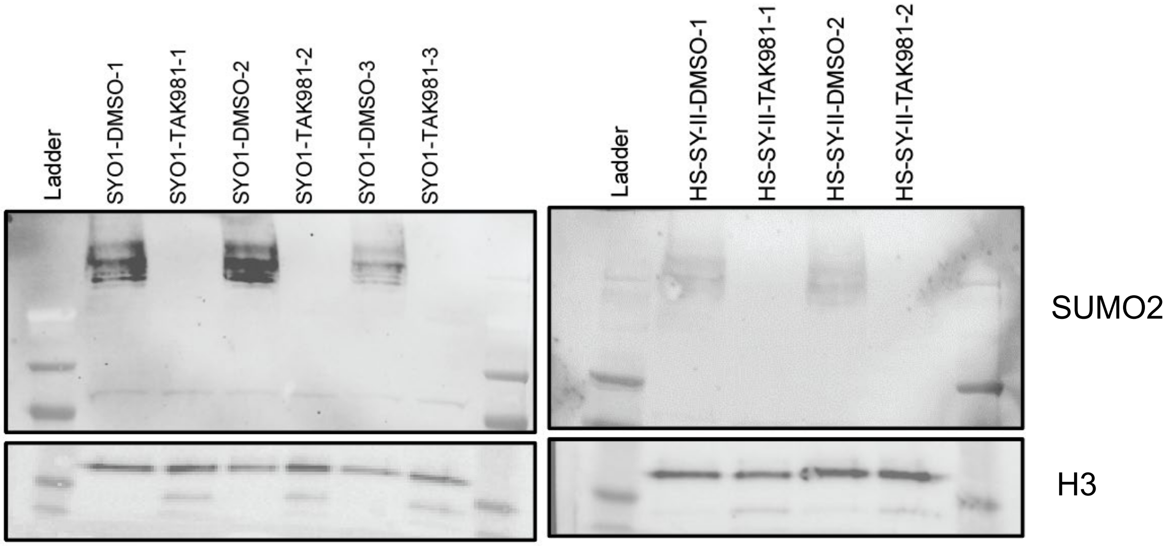

Appendix Figure S3:

Immunoblotting for total SUMO2 in lysates from SYO1 cells treated with TAK-981 compared to DMSO control are shown with Histone 3 as the loading control.

S4

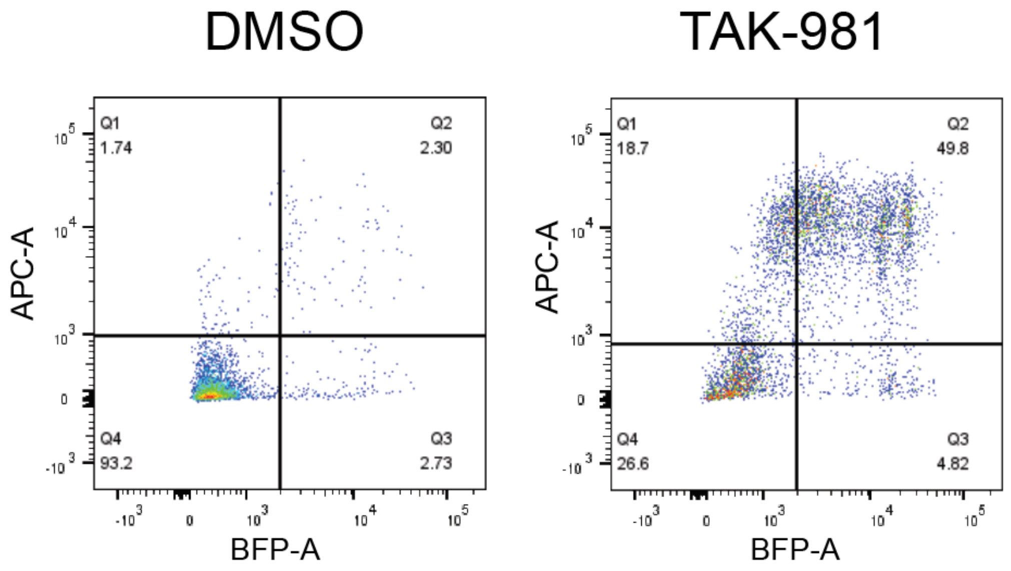

Appendix Figure S4:

Representative Flow cytometry plots to show apoptosis in SYO1 cells treated with DMSO or 1uM TAK-981 for 72 hrs. and stained with Annexin V (Y-axis) and Sytox Red (X-axis)

S5

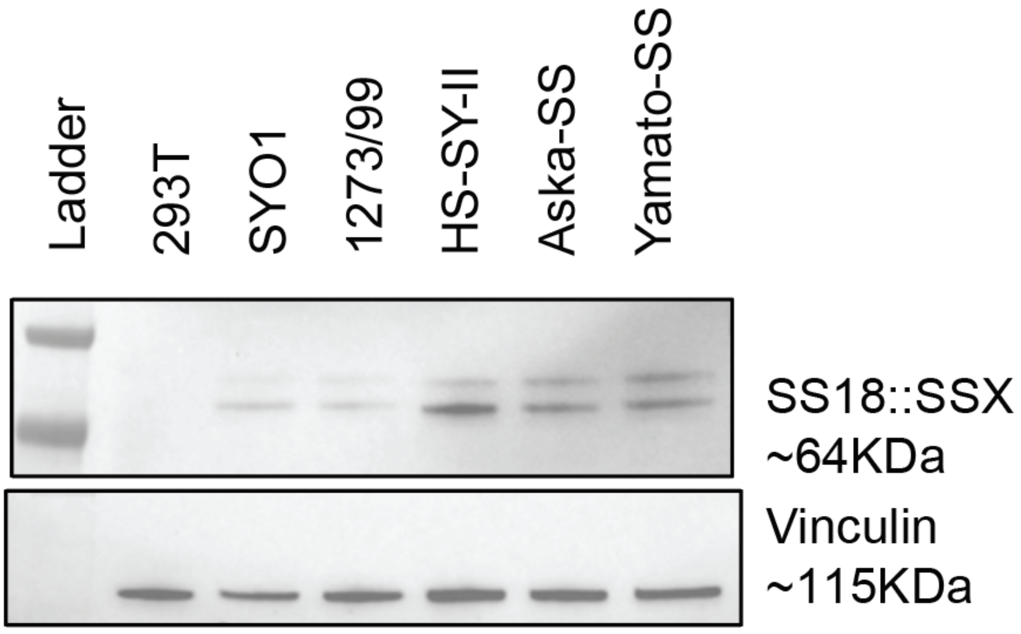

Appendix Figure S5:

Immunoblot analysis of whole cell lysates of cell lines 293T, SYO1, 1273/99, HS-SY-II, Aska-SS and Yamato-SS probed for the SS18::SSX fusion. Vinculin is shown as a loading control.

S6

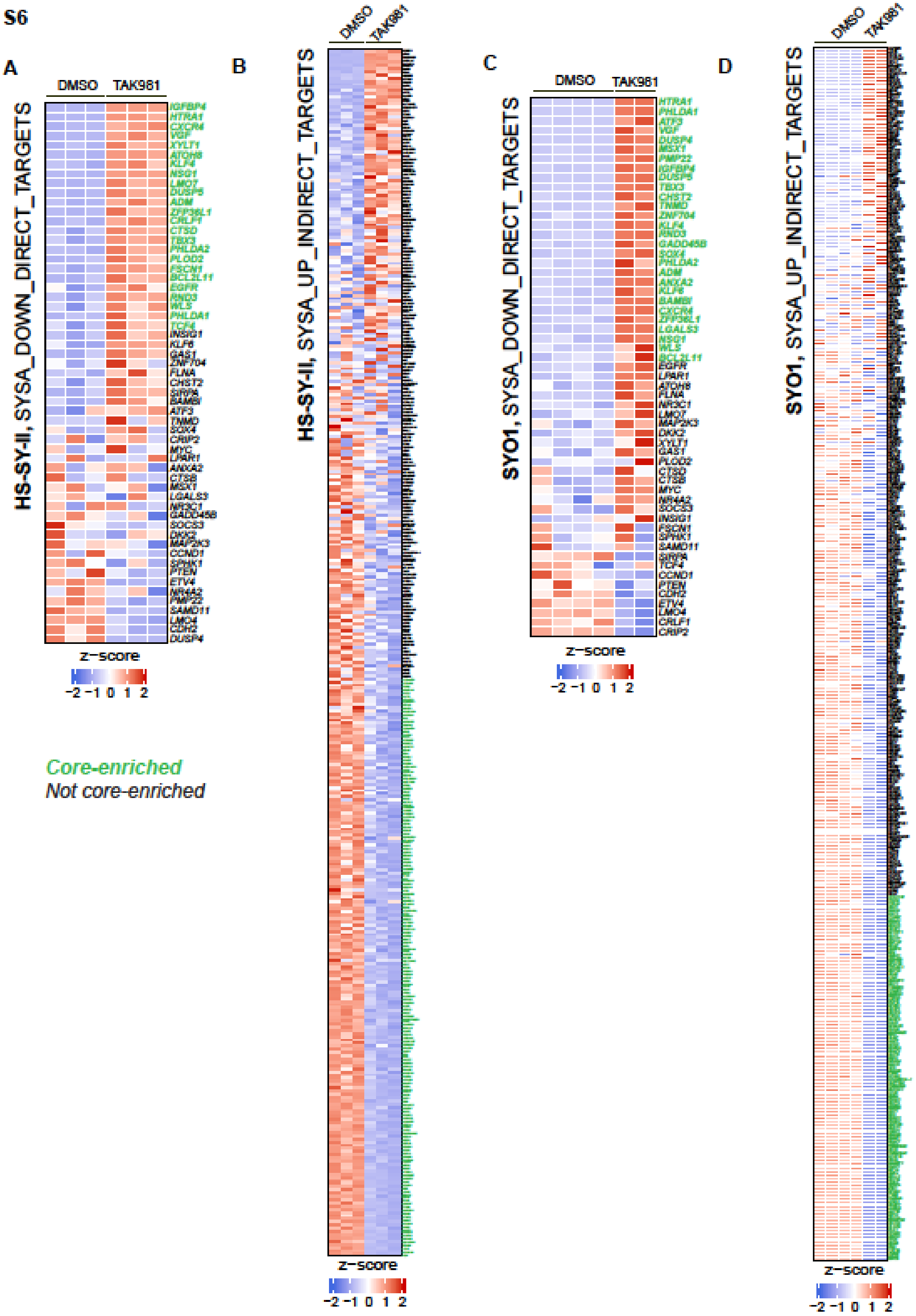
A
DMSO
TAK981
HS-SY-II, SYSA_DOWN_DIRECT_TARGETS
IGFBP4
HTRA1
CXCR4
VGF
XYLT1
ATOH8
KLF4
NSG1
LMO7
DUSP5
ADM
ZFP36L1
CRLF1
CTSD
TBX3
PHLDA2
PLOD2
FSCN1
BCL2L11
EGFR
RND3
WLS
PHLDA1
TCF4
INSIG1
KLF6
GAS1
ZNF704
FLNA
CHST2
SIRPA
BAMBI
ATF3
TNMD
SOX4
CRIP2
MYC
LPAR1
ANXA2
CTSB
MSX1
LGALS3
NR3C1
GADD45B
SOCS3
DKK2
MAP2K3
CCND1
SPHK1
PTEN
ETV4
NR4A2
PMP22
SAMD11
LMO4
CDH2
DUSP4
z−score
−2 −1 0 1 2
Core-enriched
Not core-enriched
B
DMSO
TAK981
HS-SY-II, SYSA_UP_INDIRECT_TARGETS
z−score
−2 −1 0 1 2
C
DMSO
TAK981
SYO1, SYSA_DOWN_DIRECT_TARGETS
HTRA1
PHLDA1
ATF3
VGF
DUSP4
MSX1
PMP22
IGFBP4
DUSP5
TBX3
CHST2
TNMD
ZNF704
KLF4
RND3
GADD45B
SOX4
PHLDA2
ADM
ANXA2
KLF6
BAMBI
CXCR4
ZFP36L1
LGALS3
NSG1
WLS
BCL2L11
EGFR
LPAR1
ATOH8
FLNA
NR3C1
LMO7
MAP2K3
DKK2
XYLT1
GAS1
PLOD2
CTSD
CTSB
MYC
NR4A2
SOCS3
INSIG1
FSCN1
SPHK1
SAMD11
SIRPA
TCF4
CCND1
PTEN
CDH2
ETV4
LMO4
CRLF1
CRIP2
z−score
−2 −1 0 1 2
D
DMSO
TAK981
SYO1, SYSA_UP_INDIRECT_TARGETS
z−score
−2 −1 0 1 2

Appendix Figure S6:

Heatmap displaying all the SS18-SSX fusion-activated genes shown for the SySa Down targets (A) or the SySa Up targets (B) in HS-SY-II cells or the same targets in the SYO1 cell line treated with DMSO or TAK-981. All genes are shown and the core enriched genes in the gene set enrichment analysis are marked in green.

S7

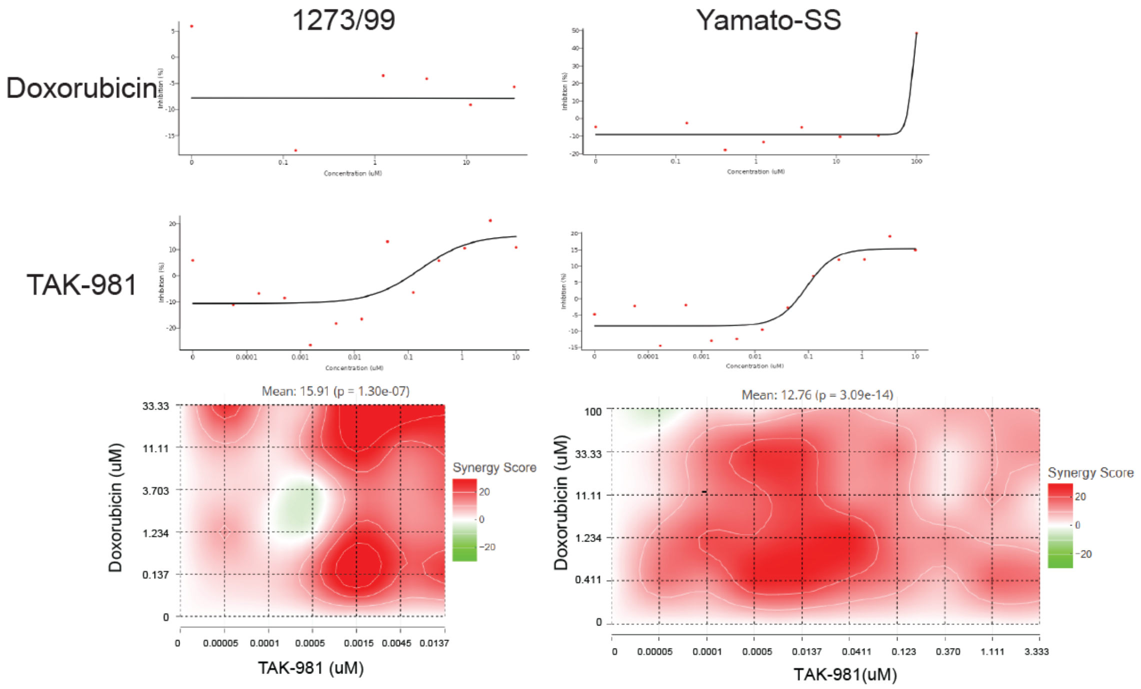

Appendix Figure S7:

IC 50 studies conducted on cell lines 1273/99 and Yamato-SS using Cell-Titer-Glo are shown. IC 50 for Doxorubicin (top), TAK-981 (middle) and a contour plot illustrating the IC 50 combination of the two drugs is shown (bottom).

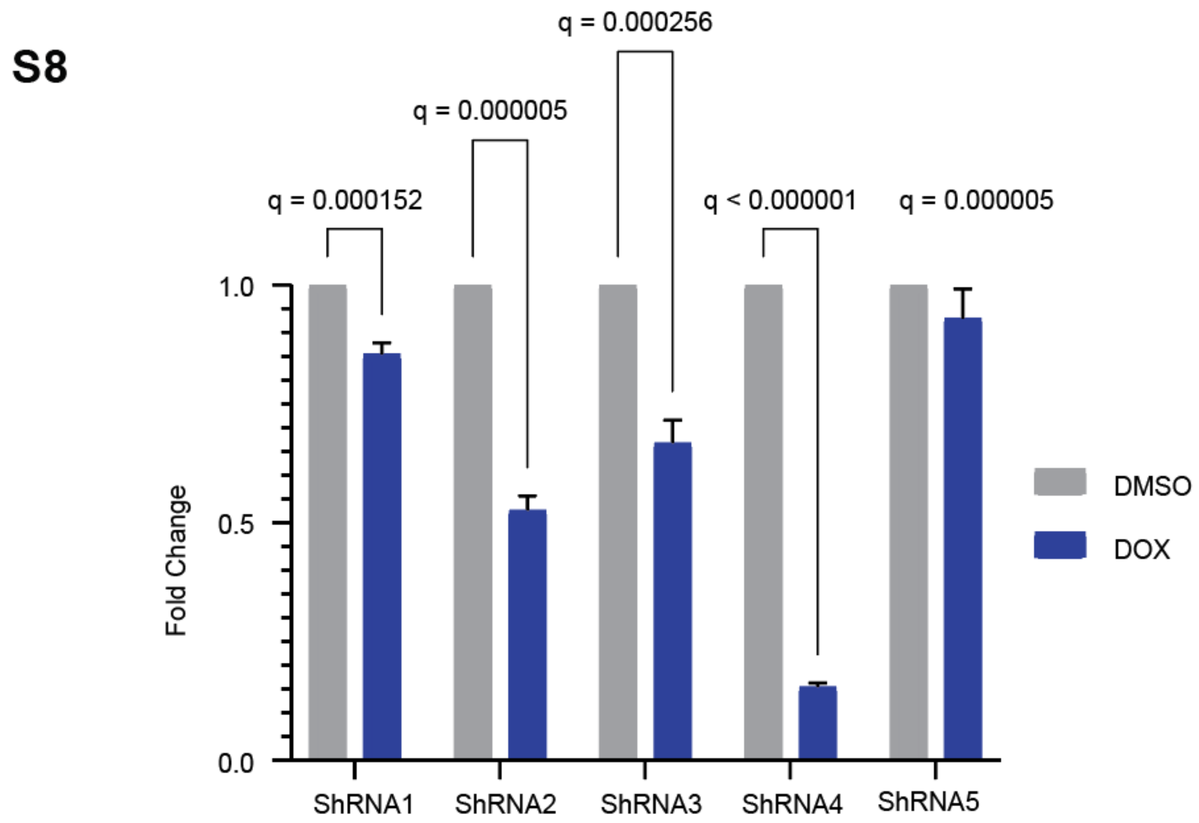

Appendix Figure S8:

Fold changes (Y-axis) of SUMO2 transcripts in doxycycline-treated relative to DMSO-treated HS-SY-II cells as measured by quantitative PCR are shown with 5 independent SUMO2-targeting shRNAs is shown (X-axis). Doxycycline was used to induce the shRNA from a tetracycline-responsive promoter.

S9

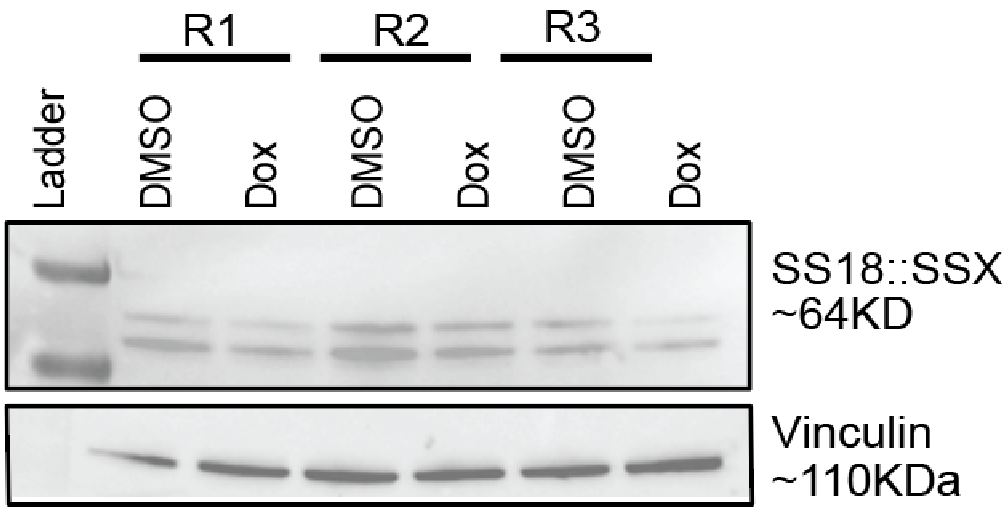

Appendix Figure S9:

Immunoblot analysis of whole-cell lysates from SYO1 cells harboring SUMO2-targeting shRNA2 and probed for SS18::SSX fusion is shown. Vinculin is used as a loading control. N = 3 biological replicates.

S10

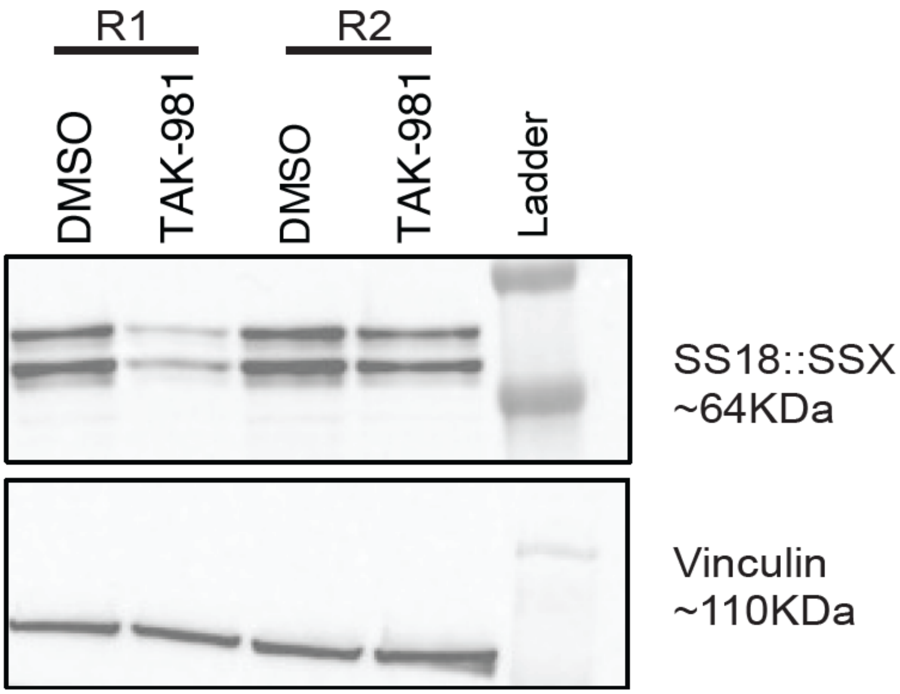

Appendix Figure S10:

Immunoblot analysis of whole-cell lysates from 1273/99 cells treated with TAK-981 and probed for the SS18-SSX2 fusion protein are shown. Vinculin is used as a loading control.

S11

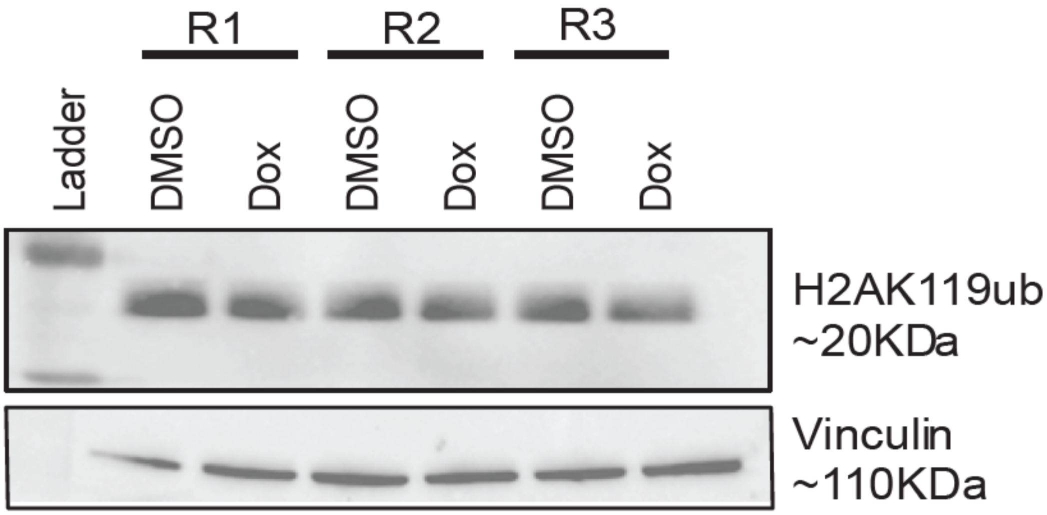

Appendix Figure S11:

Immunoblot analysis of whole-cell lysates from SYO1 cells harboring SUMO2-targeting shRNA2 and probed for H2AK119ub is shown. Vinculin from Appendix Fig S9 is used as a loading control and is redisplayed here. N = 3 biological replicates.

S12

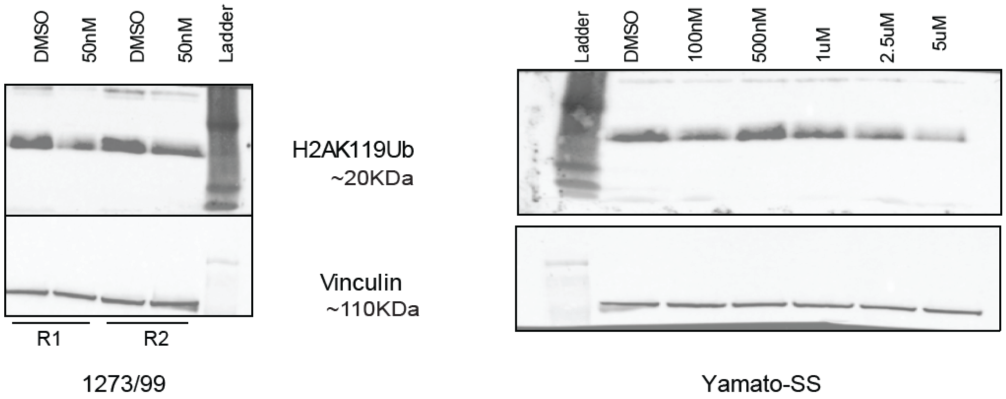

Appendix Figure S12:

Immunoblot analysis of whole-cell lysates from 1273/99 and Yamato-SS cells treated with DMSO or a range of concentrations of TAK-981 probed for H2AK119ub are shown. Vinculin from Appendix S10 is used as a loading control and is redisplayed here.

S13

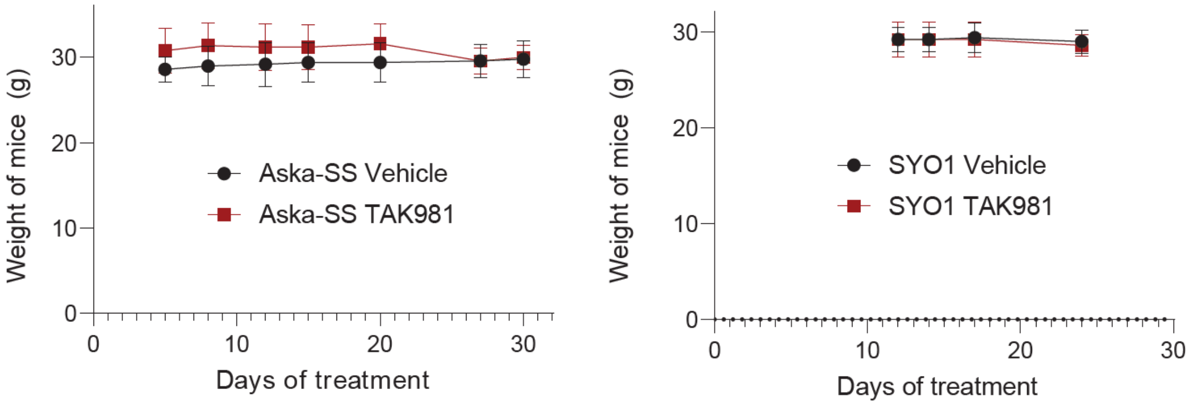

Appendix Figure S13:

Weights of mice (grams, Y-axis), treated with TAK-981 (scarlet) compared to DMSO (black) lines are shown at various time intervals (days, X-axis).
