# Supplementary figures and images for: Targeting SUMO2 reverses aberrant epigenetic rewiring driven by SS18::SSX fusion oncoproteins and impairs sarcomagenesis
Source: EMBO J. 2025 Aug 13;44(18):4984–5004. doi: 10.1038/s44318-025-00526-w (PMC12436642; doi:10.1038/s44318-025-00526-w)

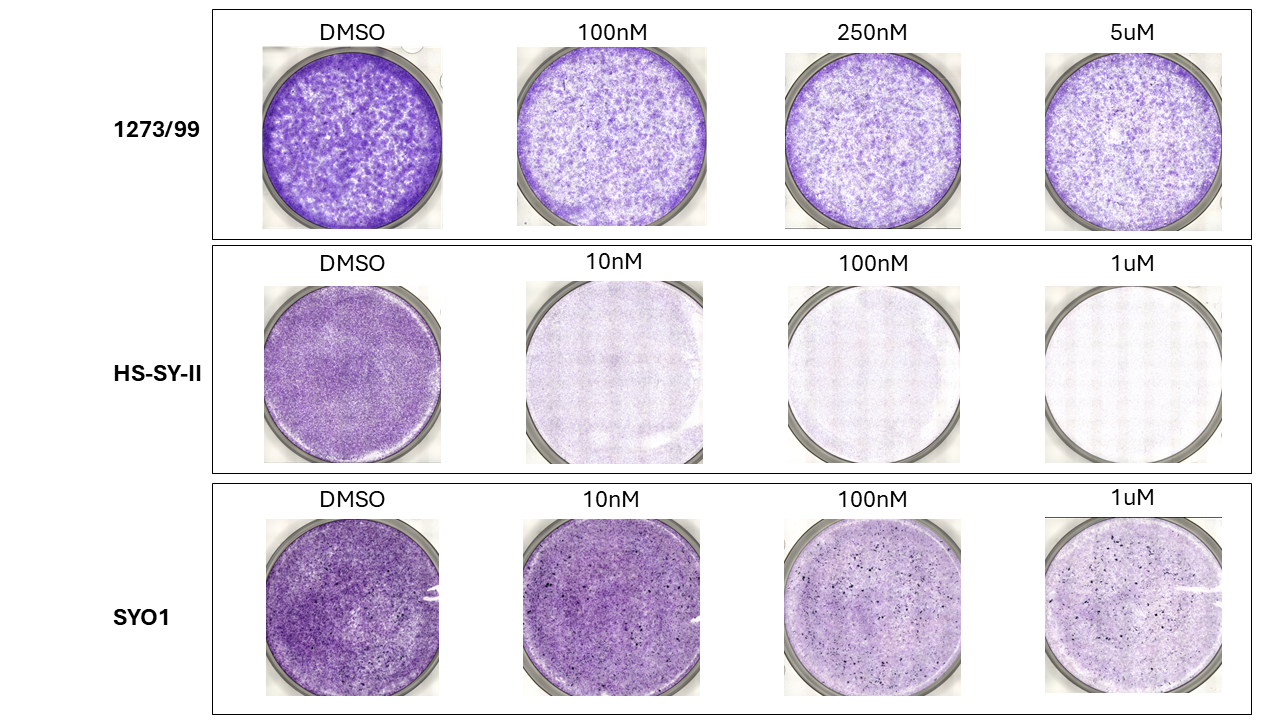

Supplement: Supplementary file 7 — Source data Fig. 3 [file 44318_2025_526_MOESM7_ESM.zip › Figure 3/3f/3f.tif]

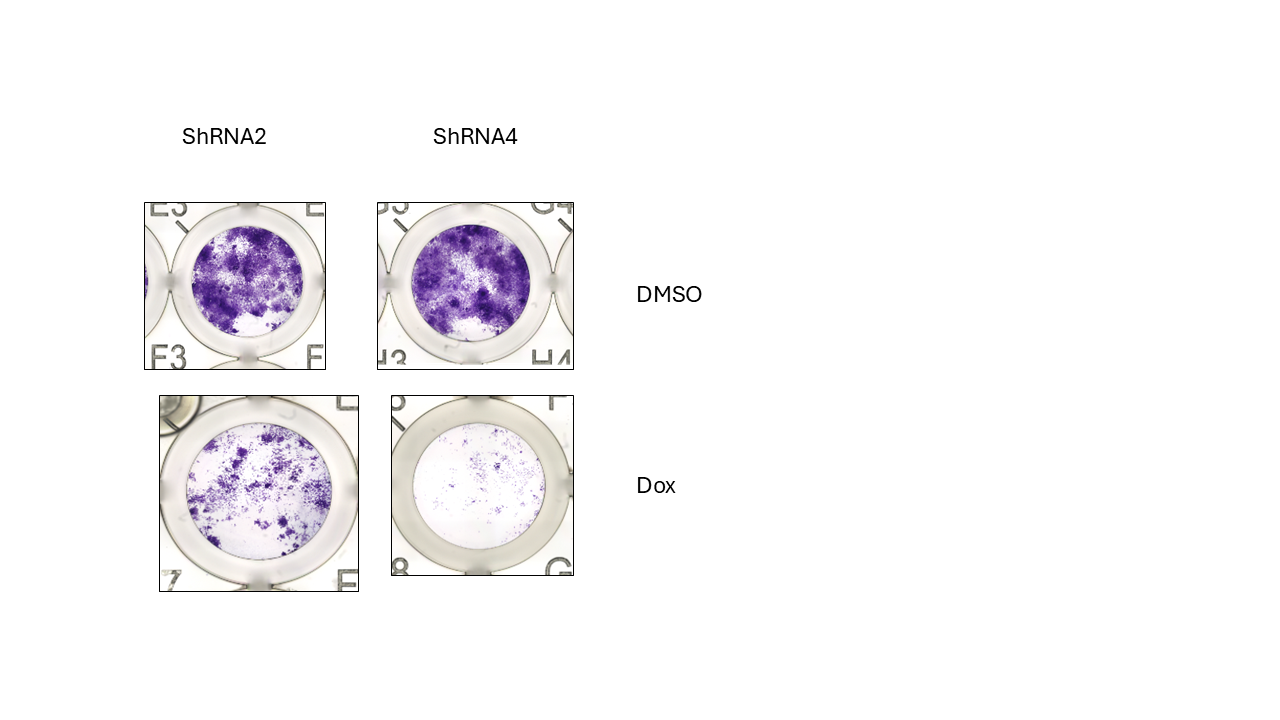

Supplement: Supplementary file 7 — Source data Fig. 3 [file 44318_2025_526_MOESM7_ESM.zip › Figure 3/3h/3h_HS_SY_II.tif]

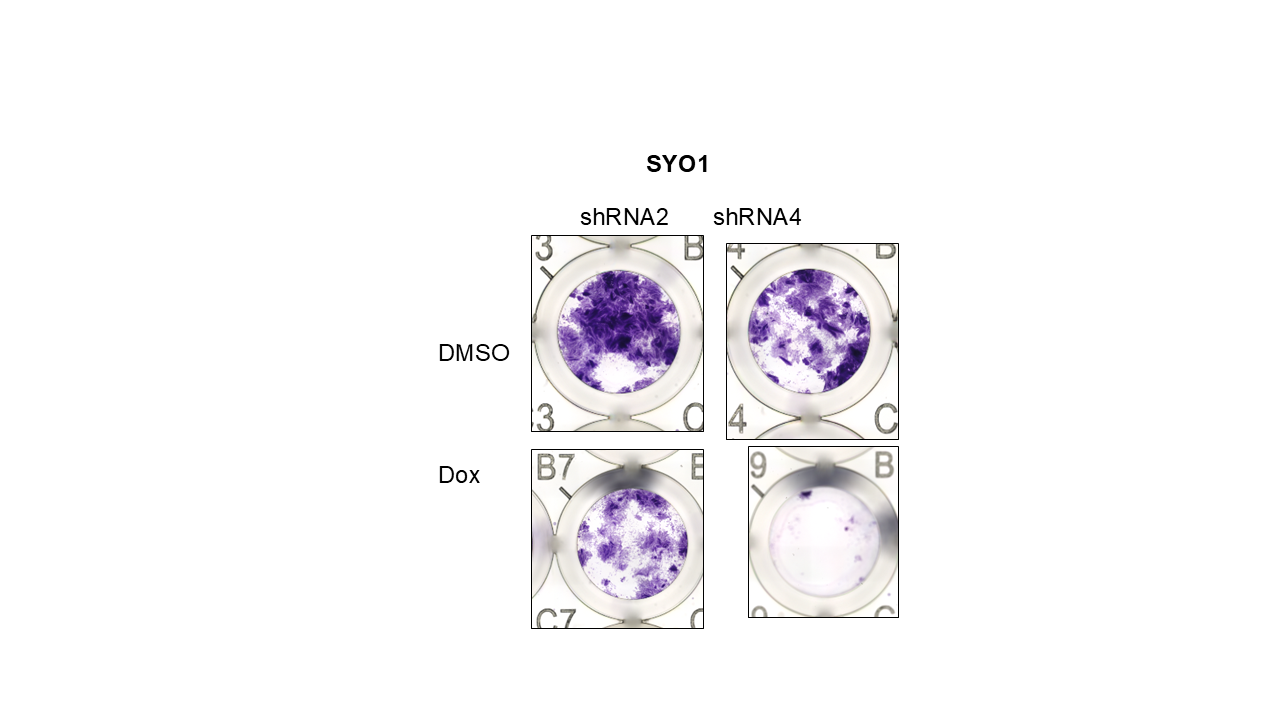

Supplement: Supplementary file 7 — Source data Fig. 3 [file 44318_2025_526_MOESM7_ESM.zip › Figure 3/3h/3h_SYO1.tif]

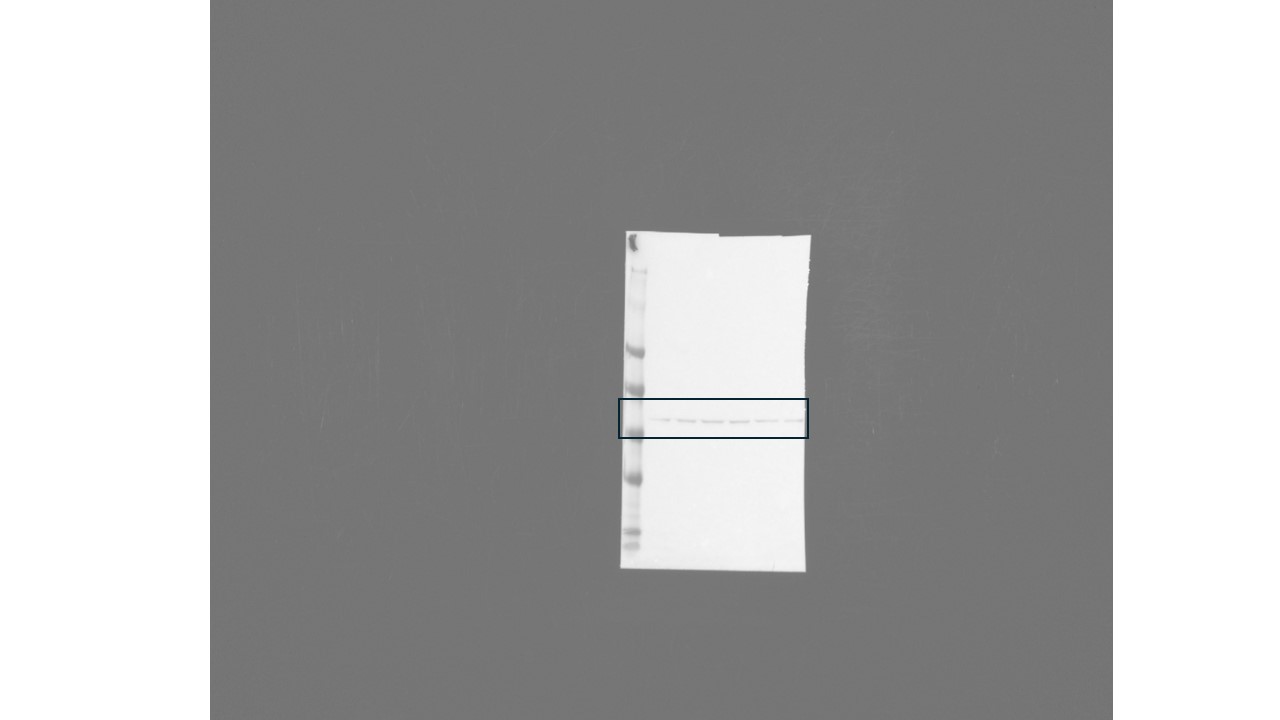

Supplement: Supplementary file 8 — Source data Fig. 5 [file 44318_2025_526_MOESM8_ESM.zip › Figure 5/5g/5g_b_actin.jpg]

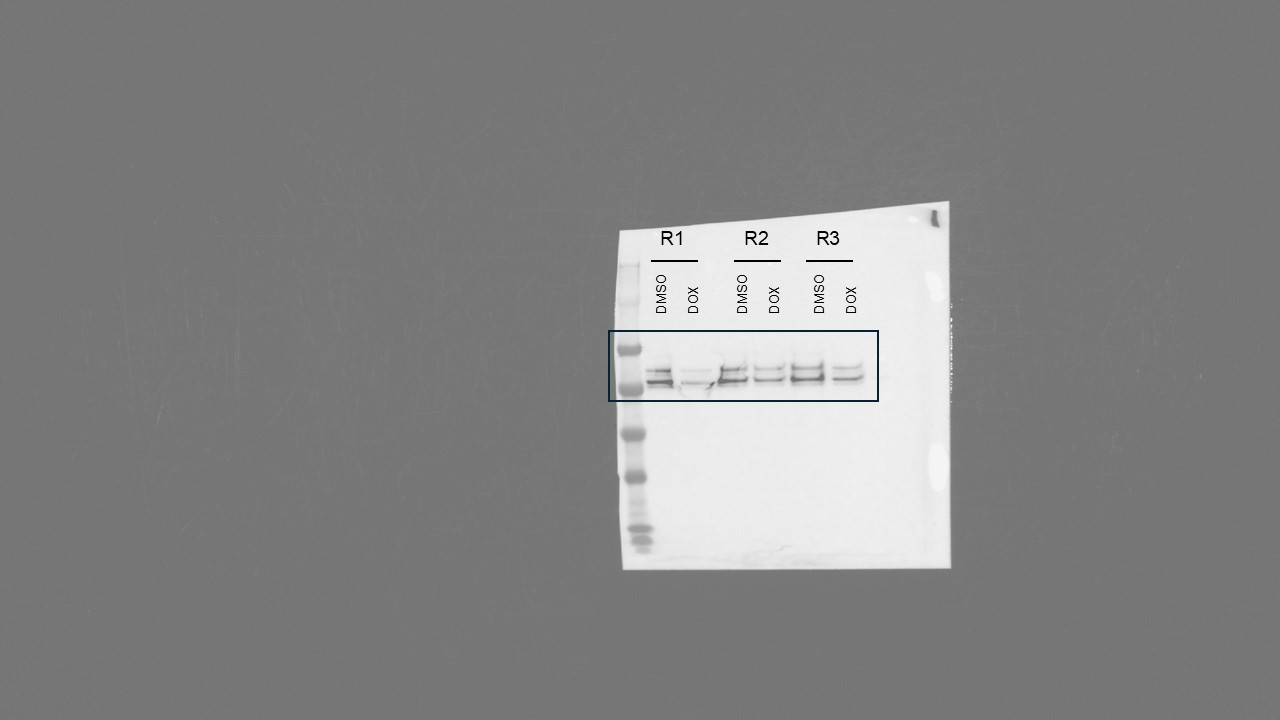

Supplement: Supplementary file 8 — Source data Fig. 5 [file 44318_2025_526_MOESM8_ESM.zip › Figure 5/5g/5g_fusion.jpg]

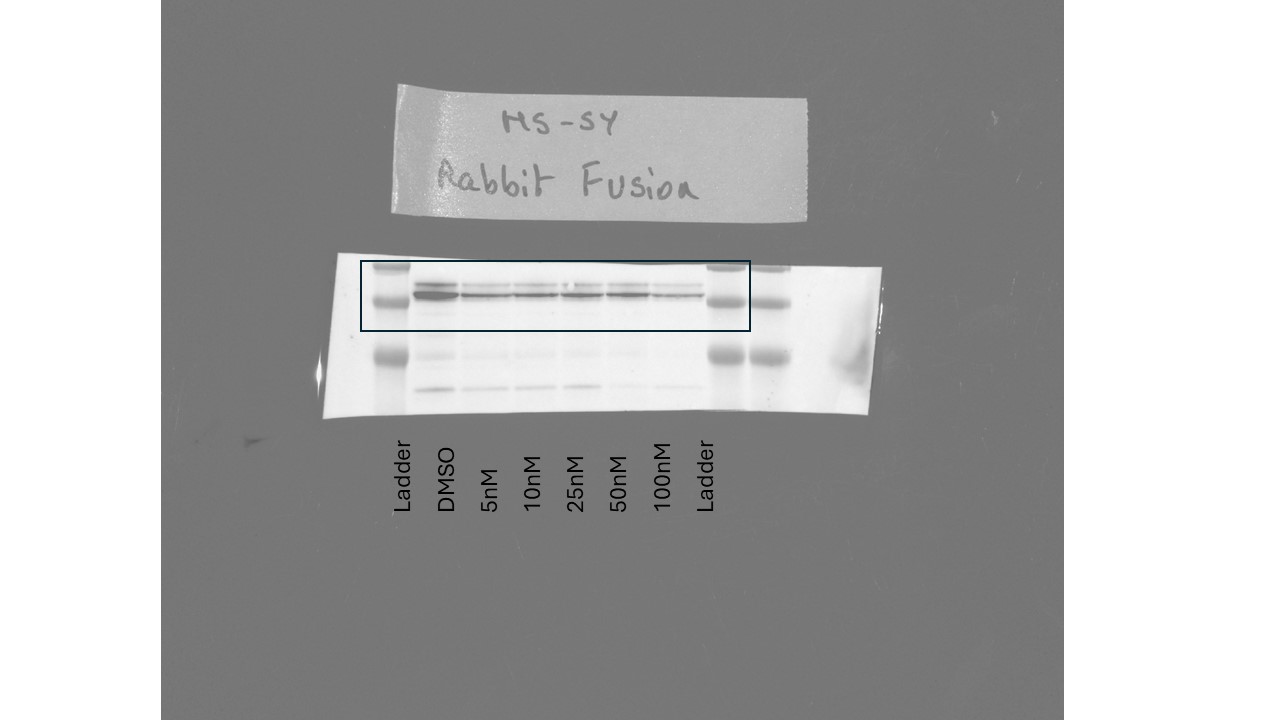

Supplement: Supplementary file 8 — Source data Fig. 5 [file 44318_2025_526_MOESM8_ESM.zip › Figure 5/5h/5h_fusion.jpg]

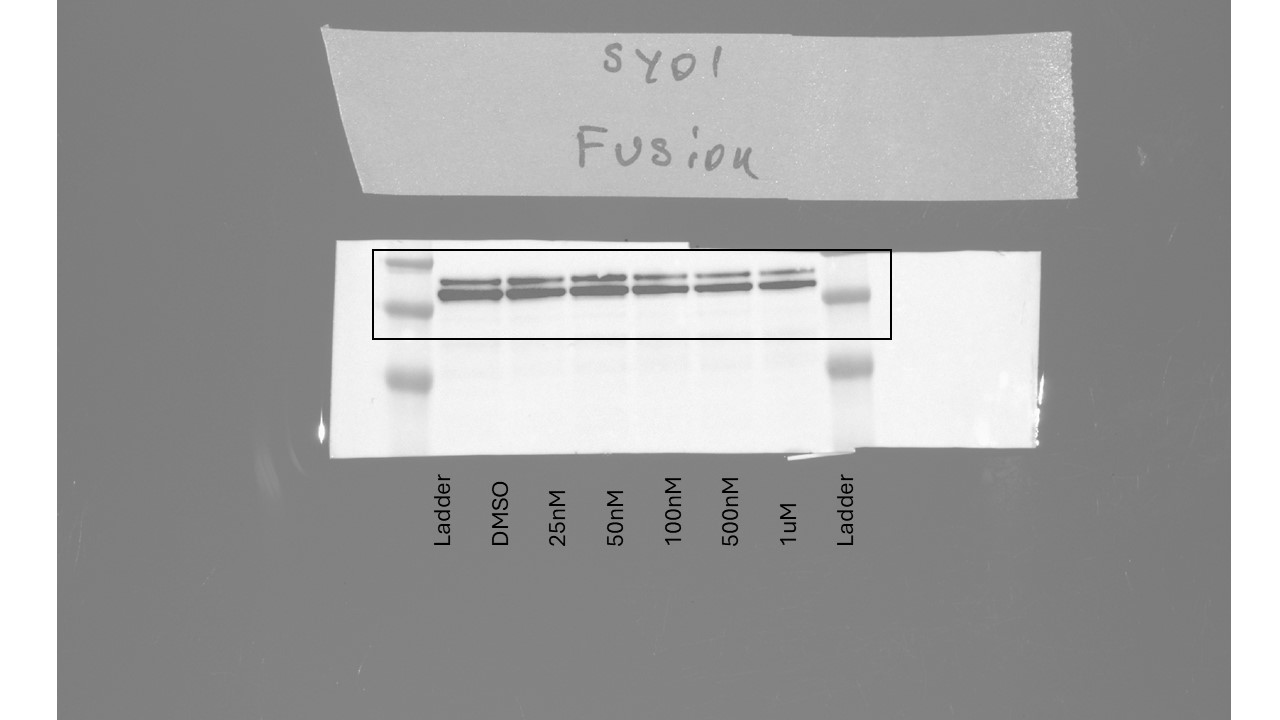

Supplement: Supplementary file 8 — Source data Fig. 5 [file 44318_2025_526_MOESM8_ESM.zip › Figure 5/5i/5i.jpg]

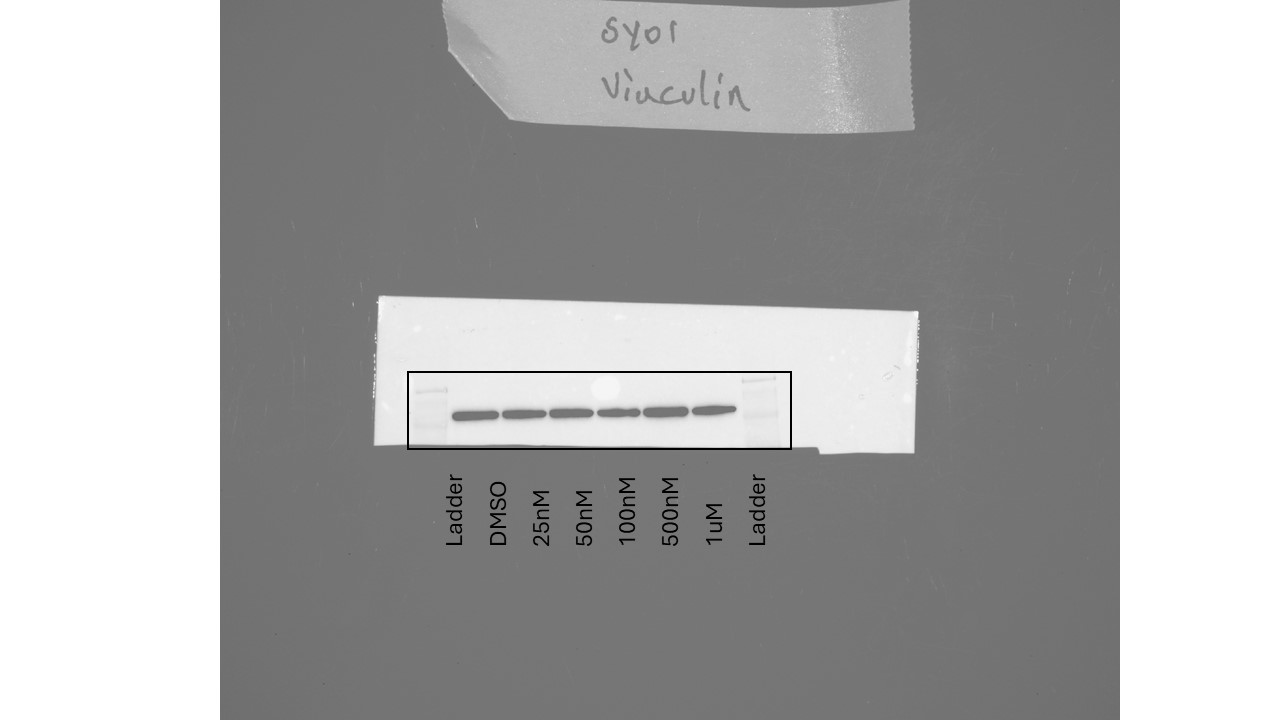

Supplement: Supplementary file 8 — Source data Fig. 5 [file 44318_2025_526_MOESM8_ESM.zip › Figure 5/5i/5i_vinculin.jpg]

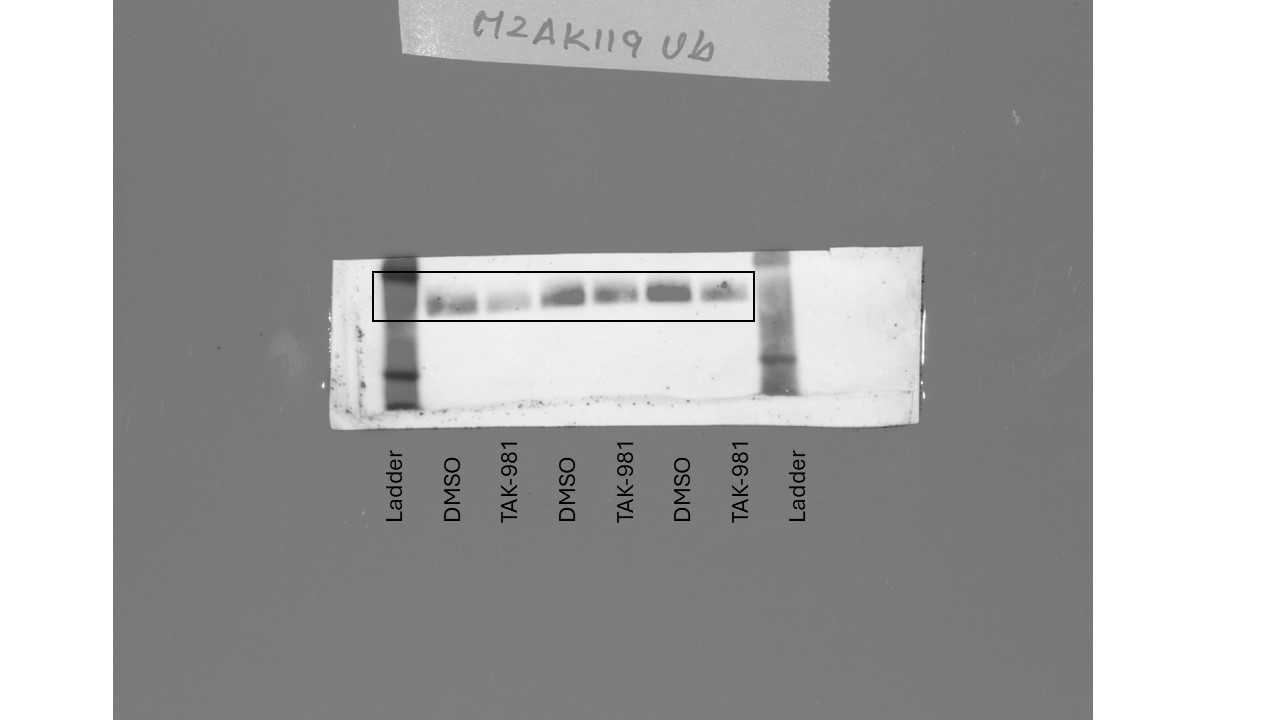

Supplement: Supplementary file 9 — Source data Fig. 6 [file 44318_2025_526_MOESM9_ESM.zip › Figure 6/6d/6d_H2AK119ub.jpg]

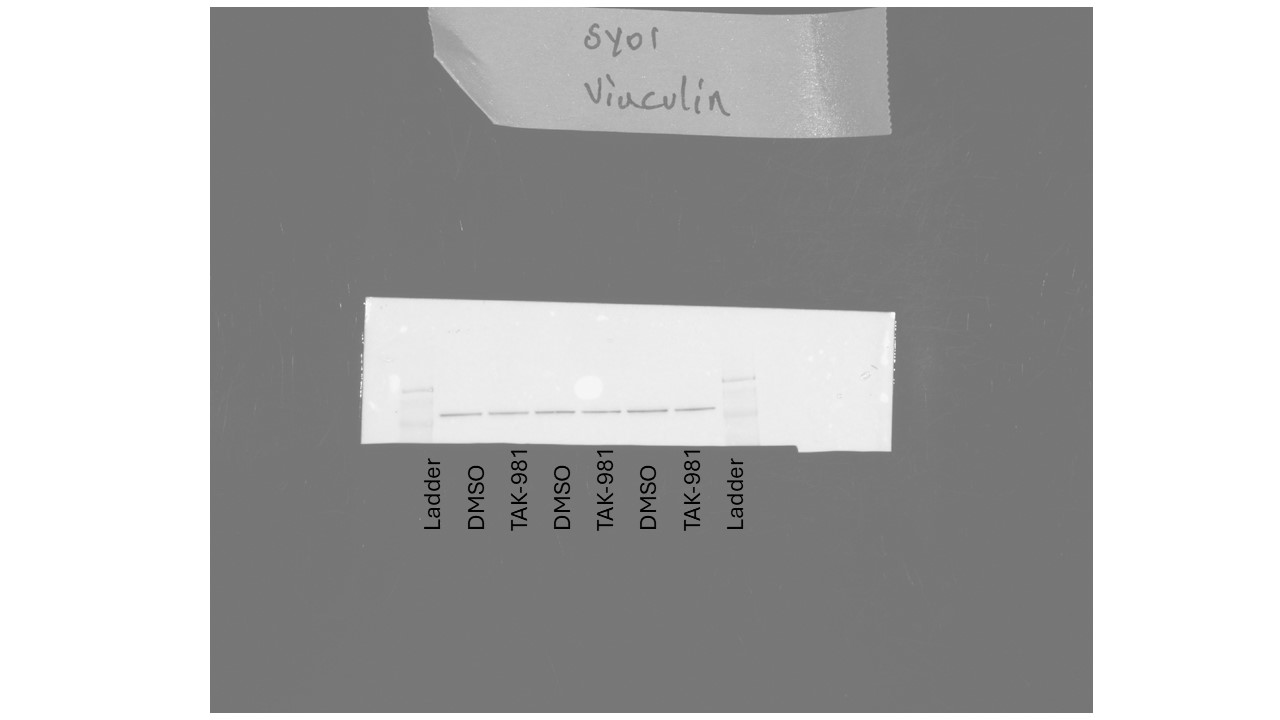

Supplement: Supplementary file 9 — Source data Fig. 6 [file 44318_2025_526_MOESM9_ESM.zip › Figure 6/6d/6d_Vinculin.jpg]

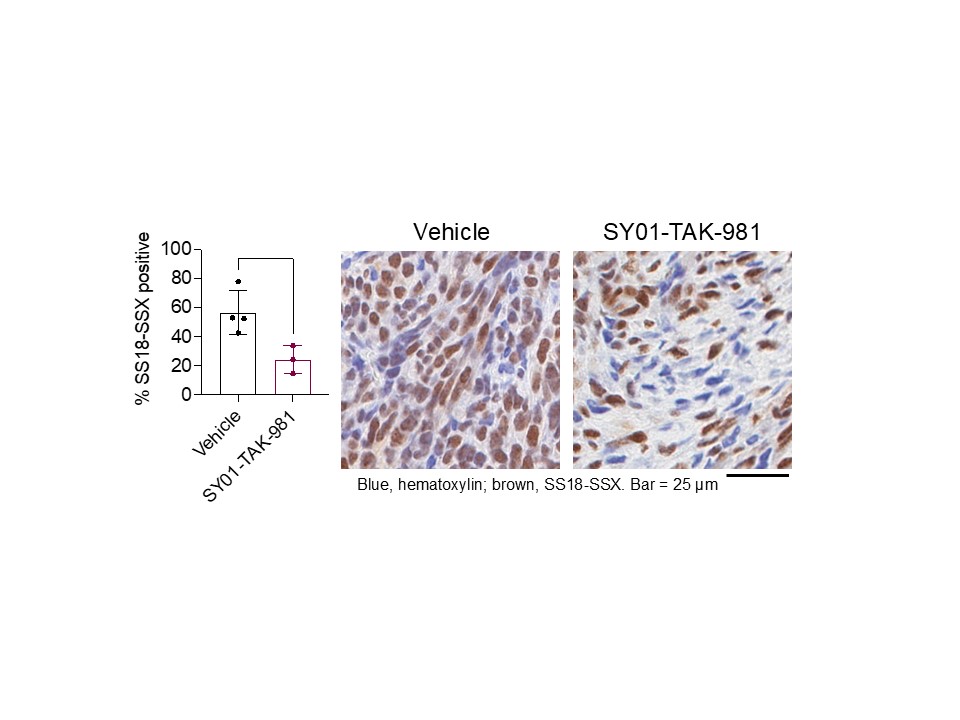

Supplement: Supplementary file 10 — Source data Fig. 7 [file 44318_2025_526_MOESM10_ESM.zip › Figure 7/7r/7r.jpg]

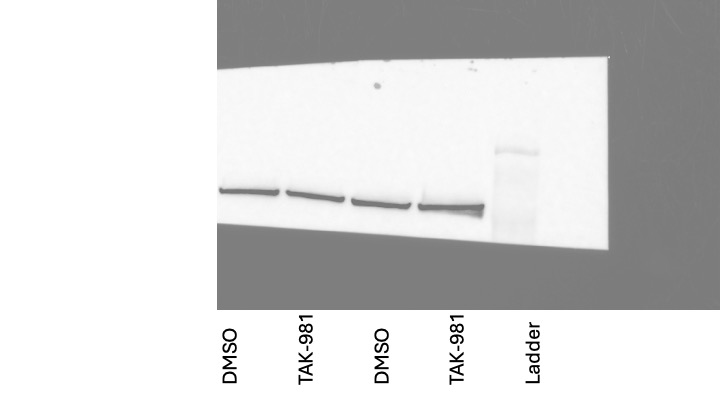

Supplement: Supplementary file 11 — Appendix Figure Source Data [file 44318_2025_526_MOESM11_ESM.zip › Supplemental Source data/S10_vinculin.jpeg]

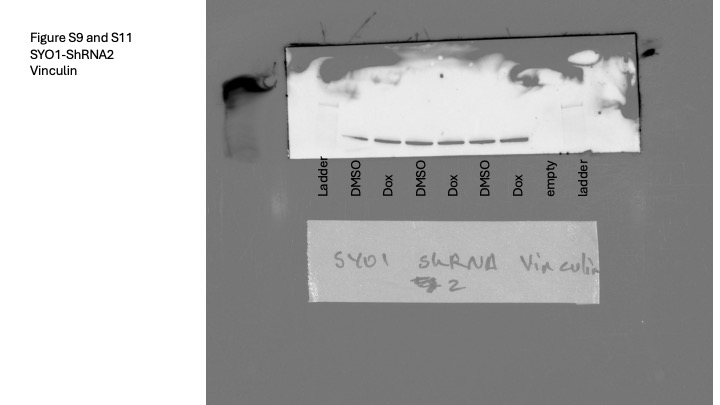

Supplement: Supplementary file 11 — Appendix Figure Source Data [file 44318_2025_526_MOESM11_ESM.zip › Supplemental Source data/S9_S11_vinculin.jpeg]

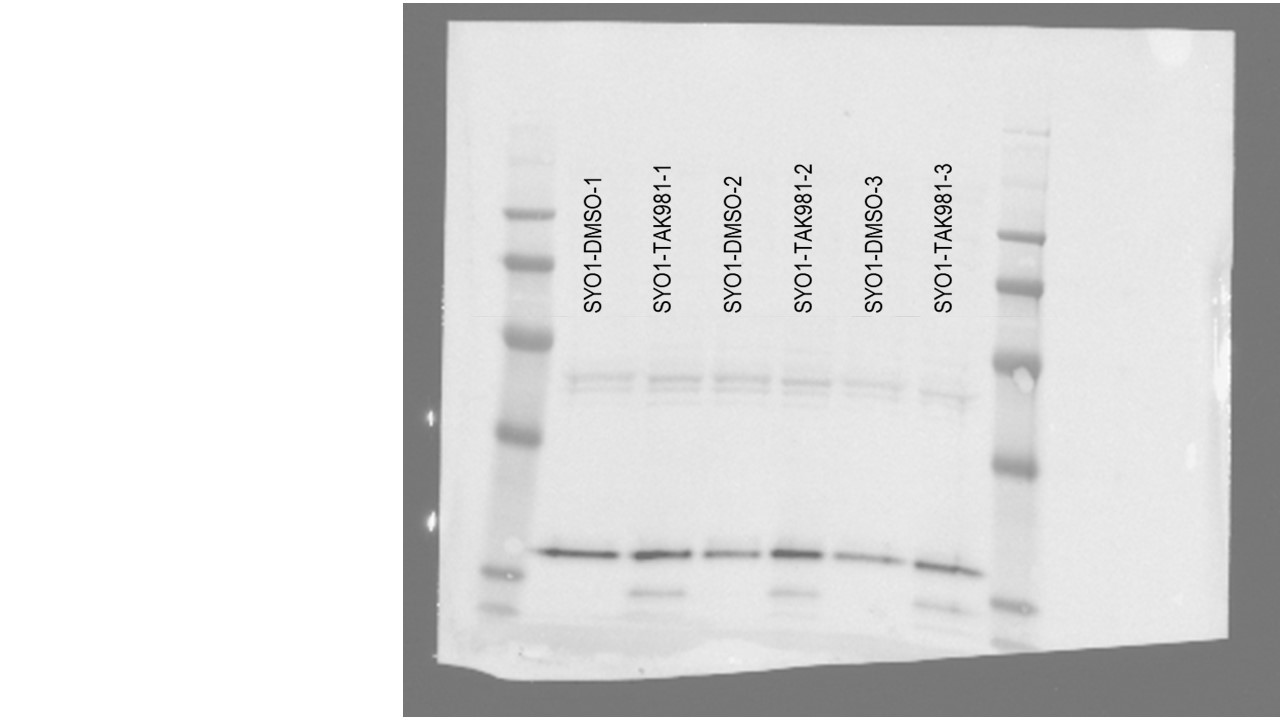

Supplement: Supplementary file 11 — Appendix Figure Source Data [file 44318_2025_526_MOESM11_ESM.zip › Supplemental Source data/S3_SYO1_H3.jpg]

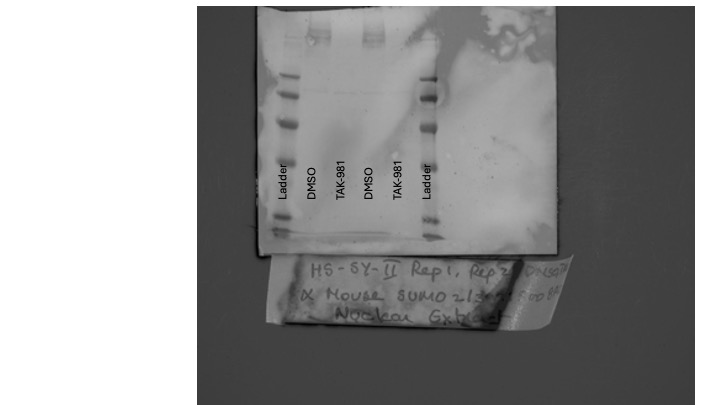

Supplement: Supplementary file 11 — Appendix Figure Source Data [file 44318_2025_526_MOESM11_ESM.zip › Supplemental Source data/S3_HS_SY_II_SUMO2.jpeg]

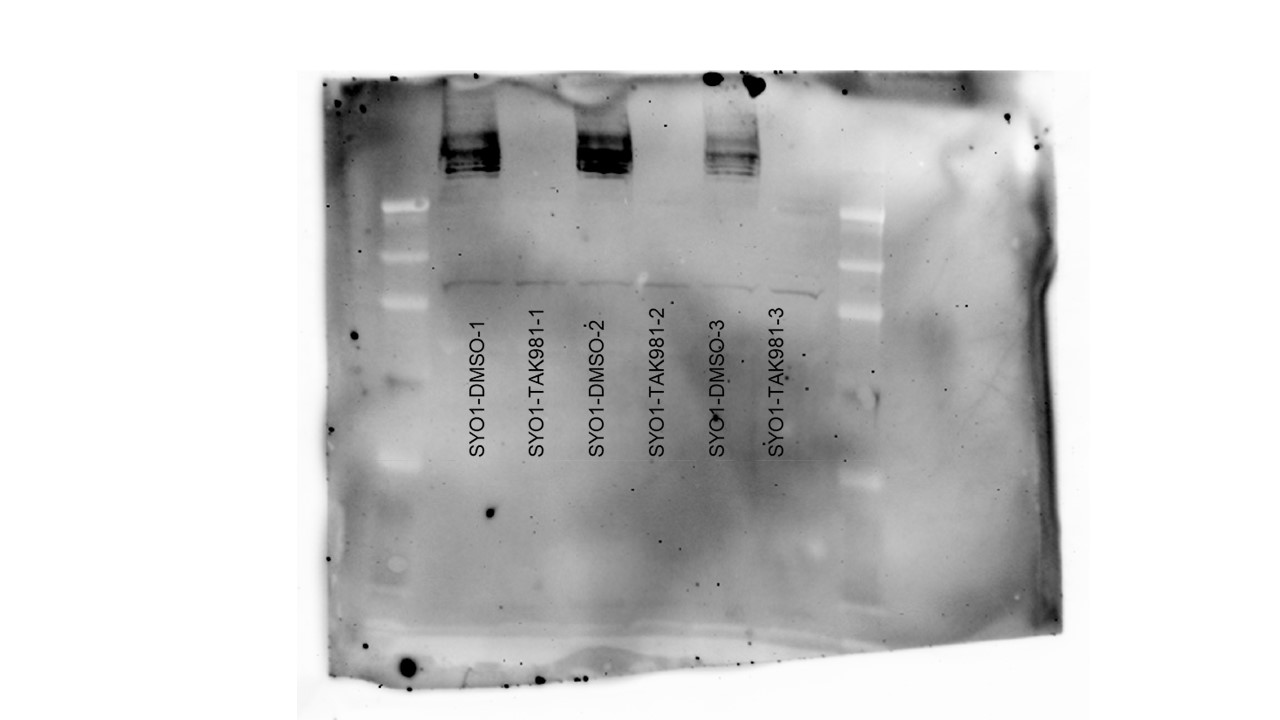

Supplement: Supplementary file 11 — Appendix Figure Source Data [file 44318_2025_526_MOESM11_ESM.zip › Supplemental Source data/S3_SYO1_SUMO2_3.jpg]

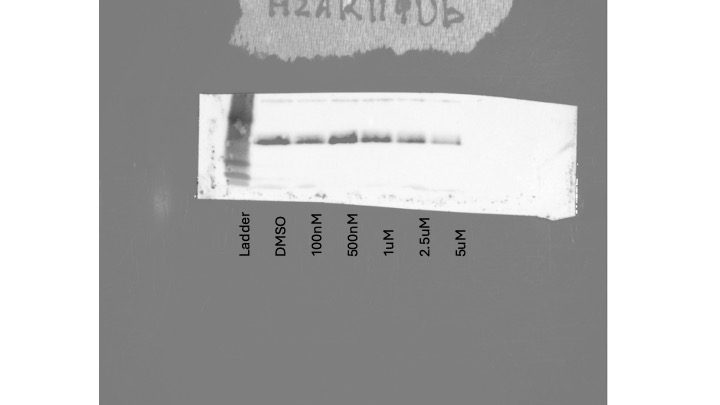

Supplement: Supplementary file 11 — Appendix Figure Source Data [file 44318_2025_526_MOESM11_ESM.zip › Supplemental Source data/S12_yamato_H2AK119ub.jpeg]

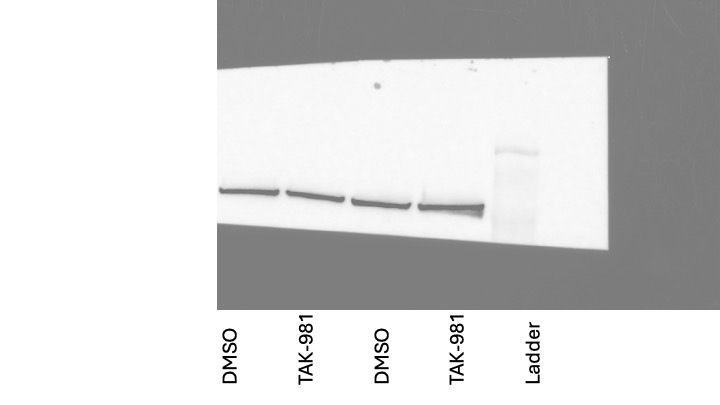

Supplement: Supplementary file 11 — Appendix Figure Source Data [file 44318_2025_526_MOESM11_ESM.zip › Supplemental Source data/S12_1273_99_Vinculin_same as S10_250722.tiff]

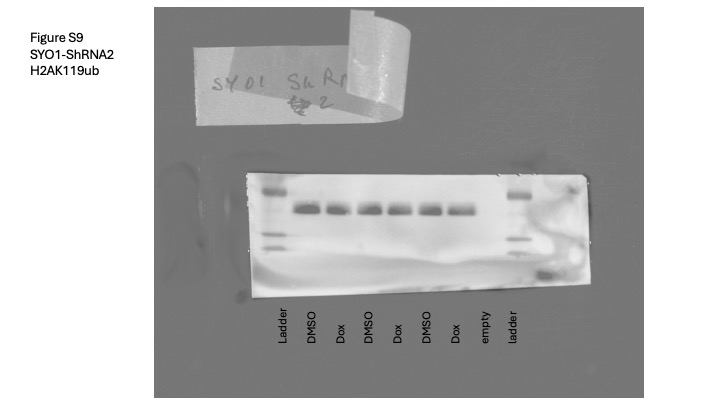

Supplement: Supplementary file 11 — Appendix Figure Source Data [file 44318_2025_526_MOESM11_ESM.zip › Supplemental Source data/S11_SYO1_ShRNA2_H2AK119ub.jpeg]

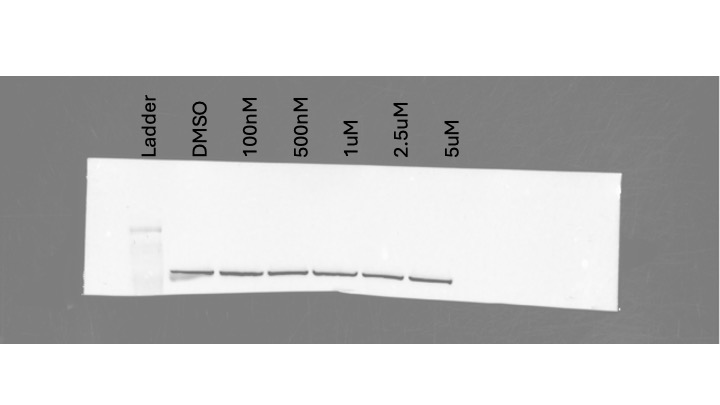

Supplement: Supplementary file 11 — Appendix Figure Source Data [file 44318_2025_526_MOESM11_ESM.zip › Supplemental Source data/S12_yamato_Vinculin.jpeg]

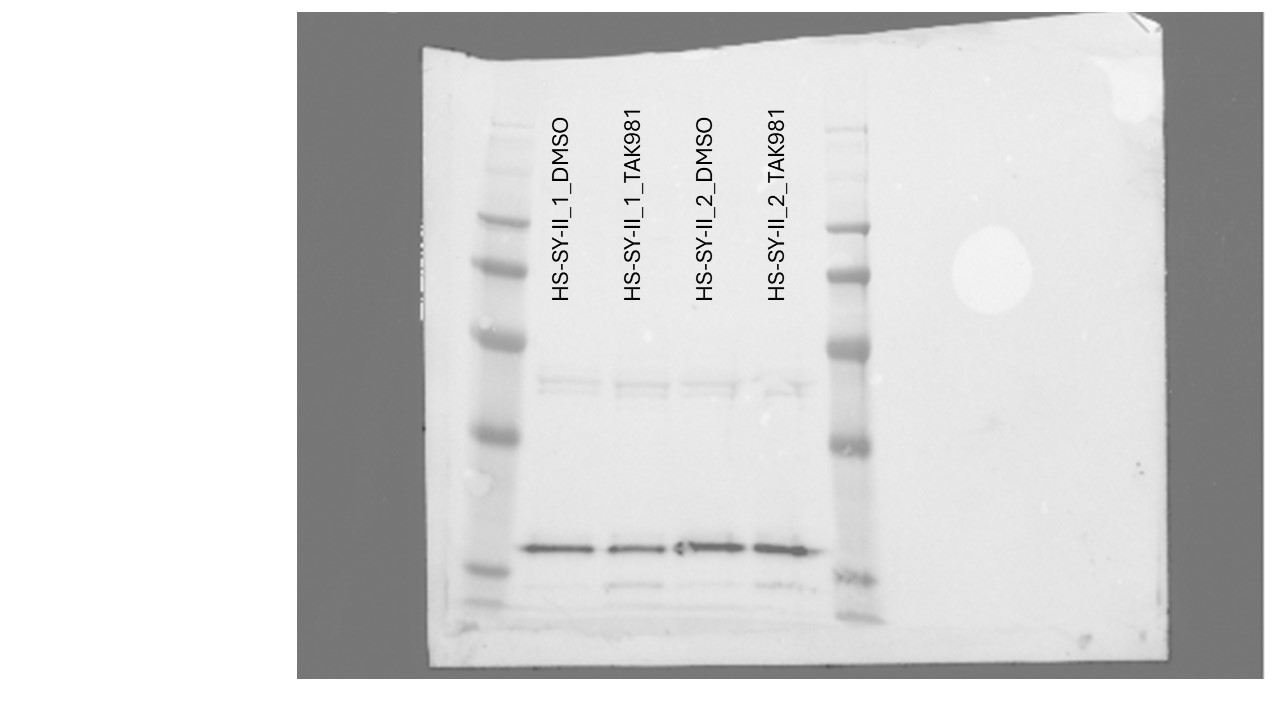

Supplement: Supplementary file 11 — Appendix Figure Source Data [file 44318_2025_526_MOESM11_ESM.zip › Supplemental Source data/S3_HS_SY_II_H3.jpg]

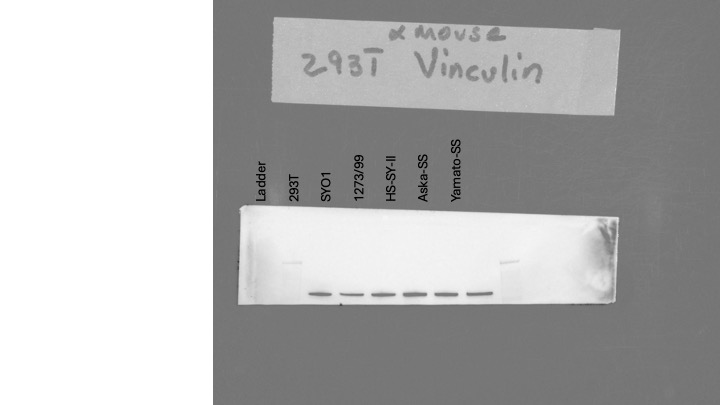

Supplement: Supplementary file 11 — Appendix Figure Source Data [file 44318_2025_526_MOESM11_ESM.zip › Supplemental Source data/S5_vinculin.jpeg]

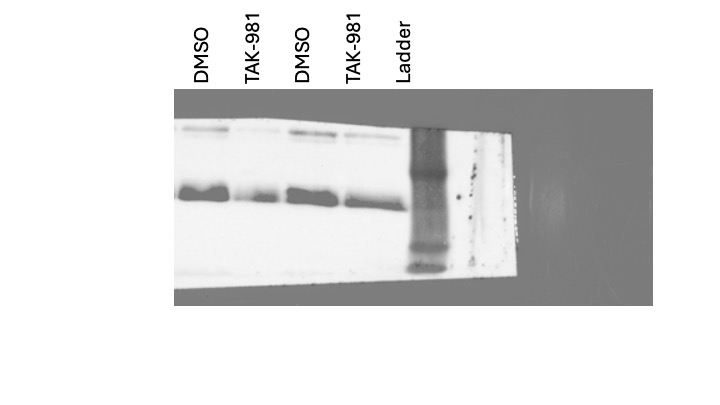

Supplement: Supplementary file 11 — Appendix Figure Source Data [file 44318_2025_526_MOESM11_ESM.zip › Supplemental Source data/S12_1273_99_H2AK119ub.jpeg]

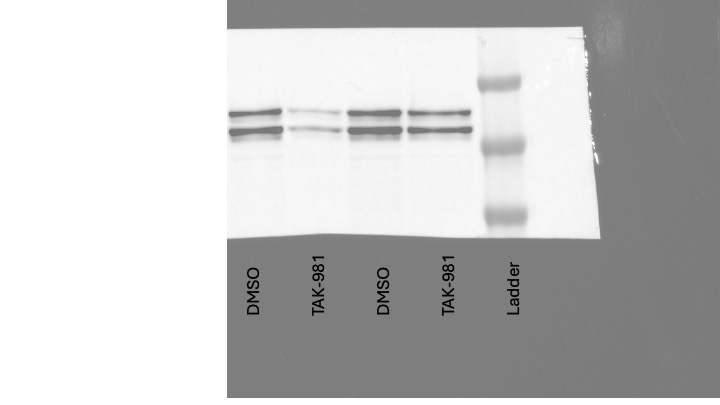

Supplement: Supplementary file 11 — Appendix Figure Source Data [file 44318_2025_526_MOESM11_ESM.zip › Supplemental Source data/S10_Fusion.jpeg]

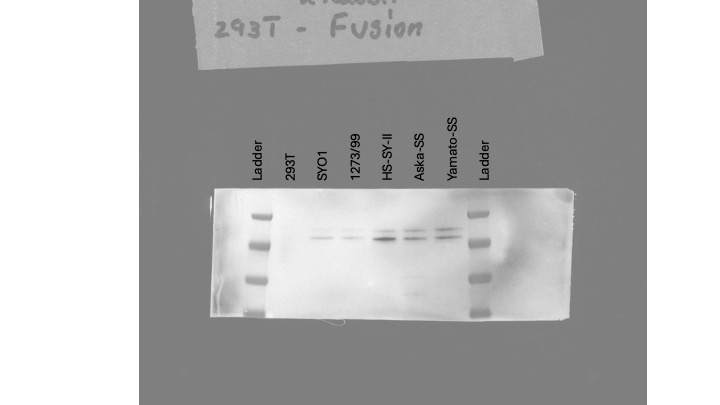

Supplement: Supplementary file 11 — Appendix Figure Source Data [file 44318_2025_526_MOESM11_ESM.zip › Supplemental Source data/S5_Fusion.jpeg]

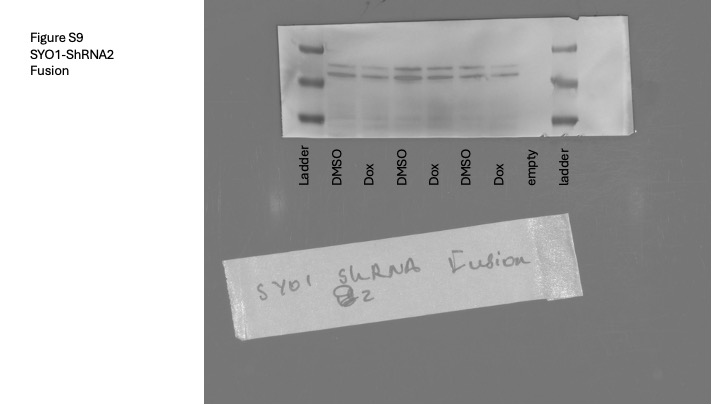

Supplement: Supplementary file 11 — Appendix Figure Source Data [file 44318_2025_526_MOESM11_ESM.zip › Supplemental Source data/S9_SYO1_ShRNA2_fusion.jpeg]
